# Impact of acute alcohol intoxication and alcohol dependence on outcomes after subarachnoid hemorrhage

**DOI:** 10.1007/s00701-025-06639-9

**Published:** 2025-08-27

**Authors:** Isaac B. Thorman, Ankita Jain, Elad Mashiach, Ariel Sacknovitz, Eris Spirollari, Rachid Kaddoura, Ruaa Alsaeed, Michael C. Schubert, Uchenna N. Okafo, Jon B. Rosenberg, Pankajavalli Ramakrishnan, Stephan A. Mayer, Chirag D. Gandhi, Fawaz Al-Mufti

**Affiliations:** 1 School of Medicine, New York Medical College, Valhalla, NY USA; 2 Johns Hopkins University School of Medicine, Baltimore, MD USA; 3 Department of Neurosurgery, Westchester Medical Center, Valhalla, NY USA; 4 Department of Neurology, Westchester Medical Center, Valhalla, NY USA; 5 College of Medicine, Mohammed Bin Rashid University of Medicine and Health Sciences, Dubai, United Arab Emirates

**Keywords:** Subarachnoid hemorrhage, Epidemiology, Neurology, Critical care, Neurosurgery

## Abstract

**Background:**

Non-traumatic subarachnoid hemorrhage (SAH) is most commonly caused by a ruptured aneurysm. Risk factors for rupture include hypertension, smoking, and substance use, but the relationship between alcohol use and clinical outcomes after SAH is poorly understood. The objective of this population-based, longitudinal, study is to characterize the relationships between alcohol use, alcohol dependence, and adverse clinical outcomes following SAH.

**Methods:**

Patients with alcohol use disorder (International Classification of Disease 10th Revision Diagnostic Code F10) in the TriNetX Research Network were compared to patients with no substance use disorders (None of F10-F19). Short-term (30-day) outcomes were assessed among patients with blood alcohol concentrations tested on the day of SAH. Outcome frequencies and Cox proportional hazard models used propensity score matching on demographics, comorbidities, blood counts, substance use, and SAH severity.

**Results:**

We identified 216,894 patients with non-traumatic SAH. Of these, 11,648 were tested for alcohol and 27,079 patients had alcohol use disorder. Blood alcohol levels of 1–100 mg/dL and above at the time of SAH were associated with decreased 30-day mortality in acute alcohol use compared to 0 mg/dL, and alcohol concentrations of 201–300 mg/dL and higher were further protective relative to 1–100 mg/dL. Patients with alcohol use disorder exhibited an increased hazard of mortality (HR = 1.175 [95% CI: 1.129, 1.223]; p < 0.0001) compared to patients with no substance use disorders (n = 151,377). Patients with severe alcohol dependence had an even higher hazard of mortality compared to patients with mild/moderate use disorder (HR = 1.139 [1.128, 1.150] p < 0.0001).

**Conclusions:**

In patients with non-traumatic SAH, alcohol in the blood at the time of SAH is protective against 30-day mortality, and increased alcohol concentration adds increased protection. Paradoxically, alcohol use disorder leads to a worsening of clinical outcomes, including mortality. There appears to be a significant dose-dependent effect of severity of alcohol dependence on mortality.

**Supplementary Information:**

The online version contains supplementary material available at 10.1007/s00701-025-06639-9.

## Introduction

Non-traumatic subarachnoid hemorrhage (SAH) is a devastating neurological event characterized by the rupture of a blood vessel or aneurysm within the brain [18]. Non-aneurysmal causes include arteriovenous malformation rupture [62], dissection of intracranial arteries [6], and amyloidopathies [31]. SAH affects 7.9 patients per 100,000 persons per year [13]. A commonly consumed substance, alcohol may promote SAH development by exacerbating hypertension, reducing platelet aggregation, and enhancing fibrinolysis [35, 44, 61, 70]. The duration and intensity of chronic alcohol use may increase the risk of SAH [12, 21–23, 27, 29, 30, 38, 53, 56, 59, 68, 71]. In contrast, some studies suggest a protective effect of occasional alcohol use against certain cardiovascular and cerebrovascular conditions, including SAH [16, 53, 71].

Prior literature has been even less consistent on the contribution of alcohol use to stroke mortality. Prospective cohort studies have focused on hypertension, cardiovascular disease, cancer, and tobacco use [2, 4, 19, 50, 65, 69]. These have largely depended on self-reported methods [2, 4, 19, 20, 41, 50, 65, 69]. A recent meta-analysis associated low alcohol use with a significantly decreased stroke mortality risk; moderate-heavy consumption had no significant association [71]. Beyond mortality, alcohol use disorder has been linked to both angiographic vasospasm and delayed cerebral ischemia, potentially due to the effects of alcohol on vascular tone [72].

While previous studies have investigated how alcohol use may modify the risk of SAH development, the relationships between alcohol use, stroke mortality, and post-hemorrhage outcomes remain poorly characterized. This longitudinal cohort study aims to elucidate the clinical outcomes of non-traumatic SAH in patients with acute alcohol use and alcohol use disorder.

## Methods

### Study Population

This study used the TriNetX Research Network to access electronic medical record data from approximately 141 million patients across 107 healthcare organizations [49]. Of these healthcare organizations, 55 are academic, 44 are non-academic, and eight are an unknown type. Data were recorded for clinical purposes, so the exact etiology and diagnostic criteria are unknown. Electronic medical record data are collected as part of routine clinical care in both inpatient and outpatient settings and are retrieved in a structured manner for demographics, diagnoses, procedures, medications, laboratory tests, and vital signs using accepted coding methodology Additionally, elements of narrative text from clinical documents are extracted using a proprietary Natural Language Processing program. This secondary data analysis does not involve interaction with human subjects and is de-identified in accordance with the de-identification standard defined in Section §164.514(a) of the Health Insurance Portability and Accountability Act Privacy Rule. This study is therefore exempt from Institutional Review Board/ethics committee approval and individual patient consent.

Within the TriNetX Research Network, patients were included in the study if their medical record included the International Classification of Disease 10th Revision (ICD-10) Diagnostic Code I60: Non-traumatic SAH when at least 18 years of age between 9/24/2004 and 12/09/2024. Ethanol concentration on the day of SAH was quantified using a TriNetX Curated lab code (LG2036-4). Patients were classified as having an alcohol use disorder based on the ICD-10 code F10: Alcohol-related disorders. A mild-moderate disorder was coded by F10.1: alcohol abuse, while F10.2 coded a severe disorder: alcohol dependence. Patients with alcohol use disorder were compared to patients with no substance use disorders (none of F10-F19), though patients who abused other substances with alcohol were included under the “alcohol use disorder” category.

### Outcomes

The primary outcome was mortality. TriNetX collects mortality data from healthcare organization death records, billing codes, the Social Security Administration Master Death File, private obituaries, and private claims. Secondary outcomes included reversible cerebral vasospasm and vasoconstriction (as recorded by ICD-10 code I67.84), acquired hydrocephalus (G91), and cerebral ischemia (I67.82). Because hydrocephalus can be a chronic medical condition, patients who were diagnosed with hydrocephalus prior to SAH were excluded from this analysis. Outcomes were temporally assessed relative to the day of hemorrhage.

### Exposure and Potential Confounders/Covariates of Interest

Propensity score matching (1:1) was used to control for differences in demographics, comorbidities, blood counts, coagulation tests, substance use, and SAH severity between groups (see Table 1 for all matching criteria). Propensity score matching criteria were assessed in the patients’ medical records up to and including the day of SAH. A nearest-neighbor method was utilized with a caliper of 0.1 pooled standard deviations and without replacement [3]. Criteria were selected based on prior studies of risk factors for SAH and risk factors for poor outcomes following SAH [8, 13, 52, 55]. Laboratory tests were matched on the frequency of patients receiving the test and, for quantitative tests, the numeric outcome. As coagulopathies increase frequencies and adverse outcomes of SAH, coagulation tests included international normalized ratio (INR), prothrombin time (PT), and activated partial thromboplastin time (aPTT). Substance tests assessed ethanol, opiates, amphetamines, cannabinoids, cocaine, and phencyclidine (Supplemental Table 1). Functional measures (Hunt-Hess grade, modified Rankin Scale, modified Fisher scale, and National Inpatient Sample-SAH Severity Score (NIS-SSS)) are not available in the TriNetX Research Network, so SAH severity was controlled for by matching on the individual criteria of the NIS-SSS (Supplemental Table 2) [37, 67].

**Table 1:** Propensity Score Matching for Alcohol Use Disorder

Criteria	Before Matching					After Matching				
	Alcohol		No Substances		*P*-value	Alcohol		No Substances		*P*-value
	*N*	% of Cohort	*N*	% of Cohort		*N*	% of Cohort	*N*	% of Cohort	
**Demographic Characteristics**										
Age at Index	26,042	52.43 ± 15.38	144,176	59.83 ± 17.08	**<0.0001**	**25,740**	**52.47 ± 15.39**	**25,740**	**52.16 ± 15.85**	**0.0226**
Sex										
Male	18,350	70.5%	66,004	45.8%	**<0.0001**	18,062	70.2%	18,101	70.3%	0.7069
Female	6,729	25.8%	73,749	51.2%	**<0.0001**	6,716	26.1%	6,659	25.9%	0.5667
Race										
White	17,382	66.7%	89,673	62.2%	**<0.0001**	17,169	66.7%	17,103	66.4%	0.5375
Black or African American	3,656	14.0%	15,981	11.1%	**<0.0001**	3,608	14.0%	3,664	14.2%	0.4785
Unknown Race	2,921	11.2%	22,694	15.7%	**<0.0001**	2,906	11.3%	2,846	11.1%	0.4012
Asian	517	2.0%	8,221	5.7%	**<0.0001**	515	2.0%	518	2.0%	0.9249
Ethnicity										
Not Hispanic or Latino	18,976	72.9%	94,608	65.6%	**<0.0001**	18,742	72.8%	18,769	72.9%	0.7890
Hispanic or Latino	2,454	9.4%	12,111	8.4%	**<0.0001**	2,424	9.4%	2,468	9.6%	0.5084
Unknown Ethnicity	4,612	17.7%	37,457	26.0%	**<0.0001**	4,574	17.8%	4,503	17.5%	0.4116
**Comorbidity Characteristics**										
I10: Essential (primary) hypertension	958	3.7%	3,882	2.7%	**<0.0001**	870	3.4%	733	2.8%	**0.0005**
E11: Type 2 diabetes mellitus	285	1.1%	1,857	1.3%	**0.0099**	269	1.0%	217	0.8%	**0.0178**
I48: Atrial fibrillation and flutter	233	0.9%	1,409	1.0%	0.2096	218	0.8%	188	0.7%	0.1350
J45: Asthma	79	0.3%	311	0.2%	**0.0065**	74	0.3%	58	0.2%	0.1632
N18: Chronic kidney disease (CKD)	133	0.5%	932	0.6%	**0.0106**	131	0.5%	99	0.4%	**0.0345**
E66: Overweight and obesity	113	0.4%	556	0.4%	0.2518	102	0.4%	79	0.3%	0.0868
I30-I5A: Other forms of heart disease	834	3.2%	3,864	2.7%	**<0.0001**	759	2.9%	655	2.5%	**0.0050**
I20-I25: Ischemic heart diseases	304	1.2%	1,486	1.0%	**0.0466**	279	1.1%	226	0.9%	**0.0178**
I82.4: DVT	49	0.2%	270	0.2%	0.9757	46	0.2%	36	0.1%	0.2691
I26: PE	43	0.2%	228	0.2%	0.7949	42	0.2%	35	0.1%	0.4247
**Laboratory Tests**										
INR (mean ± SD)	1,989	1.21 ± 0.61	8,363	1.22 ± 0.60	0.3987	1,749	1.23 ± 0.61	1,513	1.19 ± 0.48	0.1207
INR (test frequency)	1,989	7.6%	8,363	5.8%	**<0.0001**	1,749	6.8%	1,513	5.9%	**<0.0001**
PT (mean ± SD)	1,829	13.88 ± 5.85	7,685	14.06 ± 5.97	0.2765	1,605	14.07 ± 5.87	1,393	13.65 ± 5.05	**0.0485**
PT (test frequency)	1,829	7.0%	7,685	5.3%	**<0.0001**	1,605	6.2%	1,393	5.4%	**0.0001**
aPTT (mean ± SD)	1,700	29.78 ± 10.57	7,394	28.60 ± 11.75	**0.0001**	1,484	29.99 ± 10.61	1,316	28.78 ± 11.36	**0.0027**
aPTT (test frequency)	1,700	6.5%	7,394	5.1%	**<0.0001**	1,484	5.8%	1,316	5.1%	**0.0011**
Ethanol [mass/volume] (mean ± SD)	828	362.07 ± 703.72	555	104.23 ± 329.91	**<0.0001**	533	368.27 ± 651.76	405	133.79 ± 432.49	**<0.0001**
Ethanol (test frequency)	828	3.2%	555	0.4%	**<0.0001**	533	2.1%	405	1.6%	**<0.0001**
Opiates (test frequency)	244	0.9%	466	0.3%	**<0.0001**	193	0.8%	144	0.6%	**0.0074**
Amphetamines (test frequency)	192	0.7%	469	0.3%	**<0.0001**	156	0.6%	121	0.5%	**0.0350**
Cannabinoids (test frequency)	121	0.5%	221	0.2%	**<0.0001**	95	0.4%	69	0.3%	**0.0420**
Cocaine (test frequency)	124	0.5%	323	0.2%	**<0.0001**	104	0.4%	81	0.3%	0.0903
Phencyclidine (test frequency)	105	0.4%	137	0.1%	**<0.0001**	82	0.3%	64	0.2%	0.1357
**NIS-SSS Diagnoses**										
I69: Sequelae of cerebrovascular disease	73	0.3%	369	0.3%	0.4768	71	0.3%	61	0.2%	0.3835
R40.1: Stupor	61	0.2%	202	0.1%	**0.0004**	53	0.2%	43	0.2%	0.3070
I82.4: Acute embolism and thrombosis of deep veins of lower extremity	49	0.2%	270	0.2%	0.9757	46	0.2%	36	0.1%	0.2691
H49: Paralytic strabismus	13	0.1%	84	0.1%	0.6036	13	0.1%	15	0.1%	0.7054
H57.0: Anomalies of pupillary function	25	0.1%	65	0.0%	**0.0010**	17	0.1%	13	0.1%	0.4651
I69.95: Hemiplegia and hemiparesis following unspecified cerebrovascular disease	≤10	0.0%	34	0.0%		≤10	0.0%	≤10	0.0%	
I69.92: Speech and language deficits following unspecified cerebrovascular disease	≤10	0.0%	13	0.0%		≤10	0.0%	≤10	0.0%	
I69.93: Monoplegia of upper limb following unspecified cerebrovascular disease	0	0.0%	≤10	0.0%		0	0.0%	0	0.0%	
I69.94: Monoplegia of lower limb following unspecified cerebrovascular disease	0	0.0%	≤10	0.0%		0	0.0%	0	0.0%	
I69.96: Other paralytic syndrome following unspecified cerebrovascular disease	0	0.0%	≤10	0.0%		0	0.0%	0	0.0%	
I69.998: Other sequelae following unspecified cerebrovascular disease	≤10	0.0%	25	0.0%		≤10	0.0%	≤10	0.0%	
**NIS-SSS Procedures**										
5A19: Physiological Systems/Performance/Respiratory	349	1.3%	1157	0.8%	**<0.0001**	306	1.2%	219	0.9%	**0.0001**
5A1955Z: Respiratory Ventilation, Greater than 96 Consecutive Hours	155	0.6%	527	0.4%	**<0.0001**	142	0.6%	99	0.4%	**0.0055**
0BH17EZ: Insertion of Endotracheal Airway into Trachea, Via Natural or Artificial Opening	166	0.6%	462	0.3%	**<0.0001**	141	0.5%	100	0.4%	**0.0081**
5A1945Z: Respiratory Ventilation, 24-96 Consecutive Hours	137	0.5%	490	0.3%	**<0.0001**	116	0.5%	86	0.3%	**0.0344**
2.2: Ventriculostomy	≤10	0.0%	11	0.0%		≤10	0.0%	0	0.0%	
16070: Bypass Cerebral Ventricle to Nasopharynx with Autologous Tissue Substitute, Open Approach	0	0.0%	≤10	0.0%		0	0.0%	0	0.0%	
00163J2: Bypass Cerebral Ventricle to Atrium with Synthetic Substitute, Percutaneous Approach	0	0.0%	≤10	0.0%		0	0.0%	0	0.0%	
00160J4: Bypass Cerebral Ventricle to Pleural Cavity with Synthetic Substitute, Open Approach	0	0.0%	≤10	0.0%		0	0.0%	0	0.0%	
00163J6: Bypass Cerebral Ventricle to Peritoneal Cavity with Synthetic Substitute, Percutaneous Approach	≤10	0.0%	15	0.0%		≤10	0.0%	≤10	0.0%	
16077: Bypass Cerebral Ventricle to Urinary Tract with Autologous Tissue Substitute, Open Approach	0	0.0%	≤10	0.0%		0	0.0%	0	0.0%	
16078: Bypass Cerebral Ventricle to Bone Marrow with Autologous Tissue Substitute, Open Approach	0	0.0%	11	0.0%	0.1587	0	0.0%	≤10	0.0%	

### Statistical Analyses

Statistical analyses were performed on TriNetX’s Advanced Analytics Platform using R version 3.4.4 (R Foundation for Statistical Computing, Vienna, Austria) and Python version 3.6.5 (Python Software Foundation, Centrum voor Wiskunde en Informatica, Amsterdam, The Netherlands). Statistical significance was set at α < 0.05. The Advanced Analytics Platform does not return patient counts from queries with 1–10 patients, reporting all such values as “ ≤ 10.” All models used full matching.

To determine how acute alcohol use at the time of SAH may affect outcomes, Cox proportional hazard models were used. Blood alcohol levels assessed on the day of SAH were stratified as follows: 0 mg/dL, 1–100 md/dL, 101–200 mg/dL, 201–300 mg/dL, 301–400 mg/dL, and > 400 mg/dL. Patients with each alcohol content category were compared to patients with alcohol of 0 mg/dL. These models added alcohol use disorder (F10), esophageal varices (I85), and liver failure (K72) as covariates for propensity score matching.

Cox proportional hazard models were used to compare patients with alcohol use disorder to patients with no substance use disorders. Each outcome was analyzed in a separate model. Alcohol abuse (F10.1) and alcohol dependence (F10.2) were each compared to patients with no substance use disorders. Due to variations in patient behavior over time, the “abuse” and “dependence” categories were not mutually exclusive.

To evaluate for potential survivorship and severity biases, competing risk analyses were conducted for each outcome, with mortality as a competing risk to construct cumulative incidence plots and to compute competing hazard ratios for each predictor variable. The vasospasm, hydrocephalus, cerebral infarction, and cerebral ischemia outcomes were each assessed in patients who remained alive during the timeframe defined by the study.

Because we observed an increased hazard of mortality in patients with alcohol use disorder, we assessed potential causes of mortality among patients with SAH which have been demonstrated in prior studies (Supplemental Table 3) [26, 32]. Cox proportional hazard models with propensity score matching were used as described above to ascertain the hazard of each outcome associated with alcohol use disorder. Outcomes involving chronic medical conditions were studied only among patients who did not have the outcome prior to their hemorrhage. Follow-up began on the day of SAH, and patients were followed for each outcome until the end of their medical record.

## Results

### Patient Characteristics

A total of 216,894 patients with non-traumatic SAH were identified, of which 27,079 had a history of alcohol use disorder and 156,321 had no history of substance use disorder. The other 33,494 patients were excluded due to substance use disorders relating to substances other than alcohol. Of these, 26,443 patients with and without a history of alcohol use disorder were matched. In the dose–response analyses, 19,962 patients with alcohol abuse and 11,622 patients with alcohol dependence each matched to equal numbers of appropriate controls.

Prior to matching, patients with alcohol use disorder were significantly younger (52.43 ± 15.38 vs. 59.83 ± 17.08 [mean ± SD]; p < 0.0001) and significantly more likely to identify as male sex (70.5% vs. 45.8%, p < 0.0001), White race (66.7% vs. 62.2%; p < 0.0001), and non-Hispanic or Latino ethnicity (72.9% vs. 65.6%; p < 0.0001), compared to patients with no substance use disorders (Table 1). They presented with more prevalent hypertension (3.7% vs. 2.7%; p < 0.0001), asthma (0.3% vs. 0.2%; p = 0.0065), and ischemic heart diseases (1.2% vs. 1.0%; p = 0.0466). Patients with alcohol use disorder had lower rates of comorbid diabetes (1.1% vs. 1.3%; p = 0.0099) and CKD (0.5% vs. 0.6%; p = 0.0106). No clinically significant differences in matching criteria were present after matching. After matching on criteria listed in Table 1, patients with a history of alcohol use disorder received intensive care unit-level care more frequently than patients without substance use disorders (37.3% vs. 31.4%, p < 0.0001). There were no differences in endovascular procedures (3.0% vs. 3.3%, p = 0.1612), but patients with a history of alcohol use disorder received significantly fewer procedures involving craniotomies (2.4% vs. 2.7%, p = 0.0229) and burr holes (2.1% vs. 2.6%, p < 0.0001); it is unclear whether these slight, but statistically significant, differences have clinical significance.

### Clinical Outcomes with Acute Alcohol Use

**Fig. 1:**
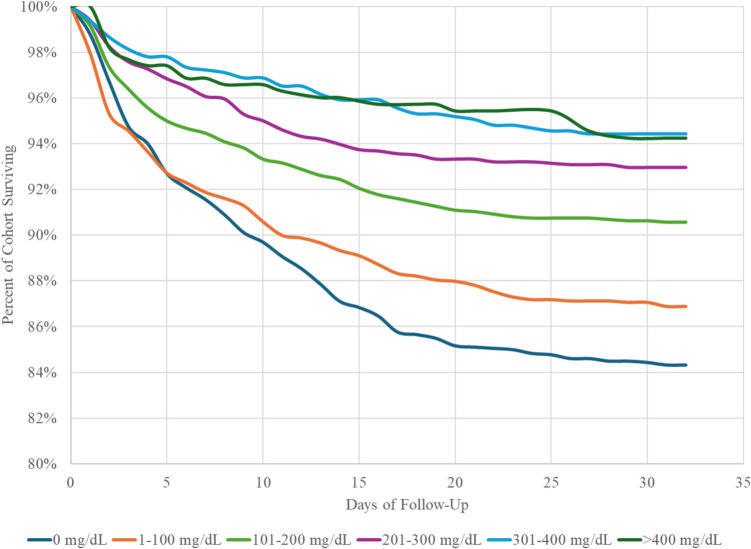
Association between alcohol use at the time of SAH and 30-day mortality

**Fig. 2:**
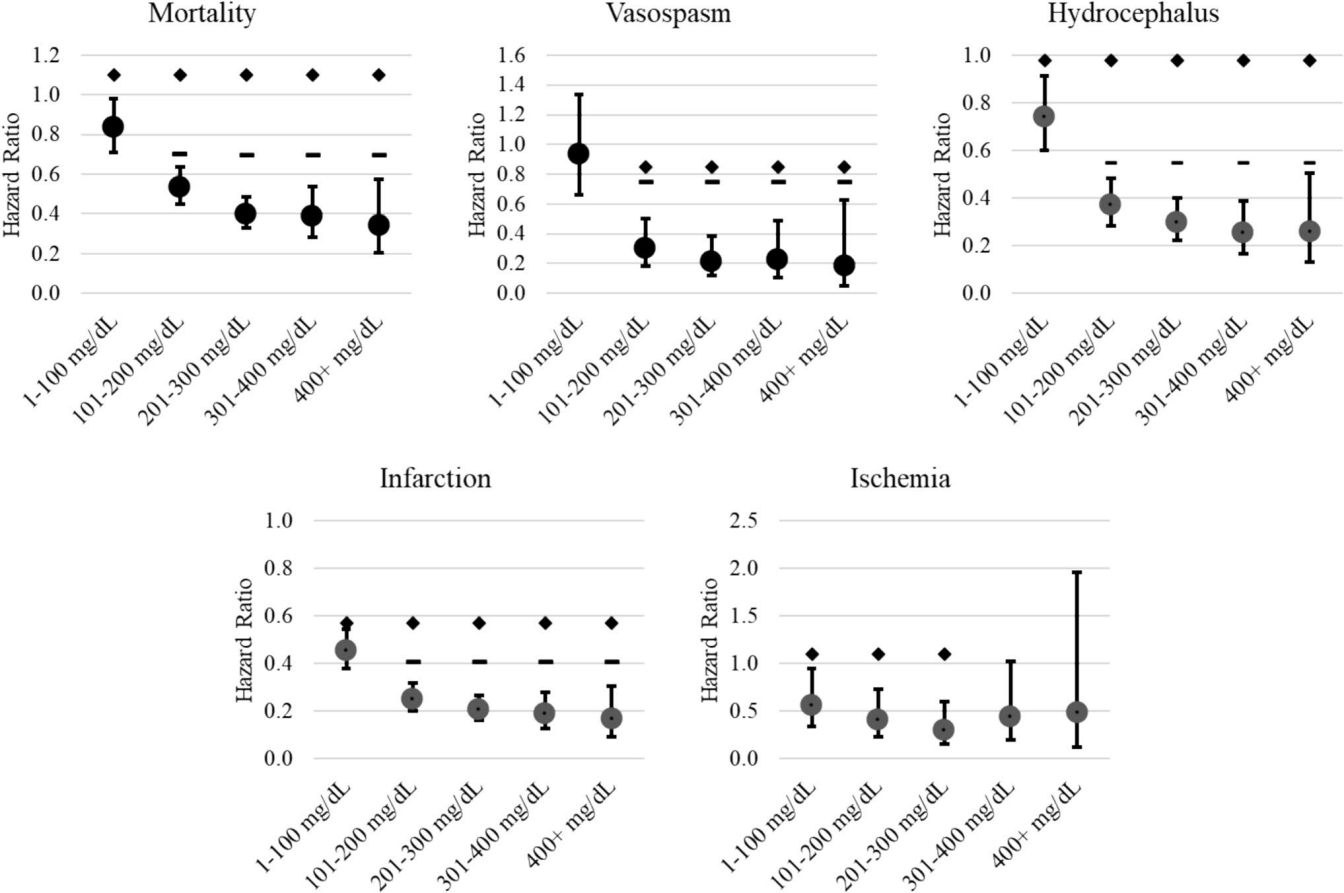
Dose-dependent protection by level of alcohol use. Hazards are relative to patients with 0 mg/dL on alcohol testing on the day of subarachnoid hemorrhage. ◊ denotes significant from 0 mg/dL;—denotes significant from 1–100 mg/dL

On the day of SAH, 11,648 patients received an ethanol test. Patients were grouped into concentrations of 0 mg/dL (20.1% of patients tested), 1–100 mg/dL (23.7%), 101–200 mg/dL (22.3%), 201–300 mg/dL (22.7%), 301–400 mg/dL (8.3%), and > 400 mg/dL (2.9%). The hazard of 30-day mortality declined with each increasing alcohol concentration (Fig. 1). Compared to a test value of 0 mg/dL, all alcohol concentrations were protective against 30-day mortality, and concentrations of 101–200 mg/dL and greater were significantly more protective than 1–100 mg/dL (Fig. 2). Ethanol concentrations of 101–200 mg/dL and greater were associated with significantly lower hazards of vasospasm than both 0 mg/dL and 1–100 mg/dL. Alcohol offered significant protection against hydrocephalus at all concentrations, and concentrations of 101–200 mg/dL and greater were associated with significantly lower hazards of hydrocephalus than 1–100 mg/dL. Alcohol use at the time of hemorrhage was significantly protective against cerebral infarction at all levels, with doses of 101–200 mg/dL being associated with significantly lower hazards of infarction than doses of 1–100 mg/dL. Alcohol use was protective against cerebral ischemia at concentrations up to and including 201–300 mg/dL.

### Clinical Outcomes of Alcohol Use Disorder

**Fig. 3:**
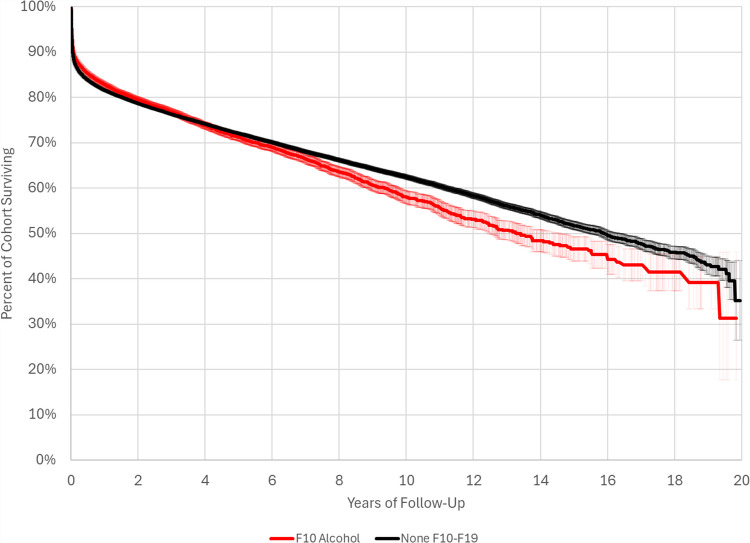
Comparative post-hemorrhage survival in alcohol use disorder and no substance use disorders

Patients with alcohol use disorder had significantly increased hazards of mortality (HR = 1.175; 95% CI: 1.129, 1.223; p < 0.0001) and cerebral ischemia (1.356; 95% CI: 1.225, 1.502; p < 0.0001), compared to patients with no substance use disorders (Fig. 3). Alcohol use disorder appeared protective against vasospasm (HR = 0.830; 95% CI: 0.769; p = 0.0001), newly-diagnosed hydrocephalus (HR = 0.769; 95% CI: 0.730; p < 0.0001), and cerebral infarction (HR = 0.943; 95% CI: 0.906, 0.983; p = 0.0014). There appeared to be a dose–response relationship, as patients with alcohol dependence (HR = 1.302; 95% CI: 1.232, 1.376; p < 0.0001) had a significantly increased hazard ratio compared to patients with alcohol abuse (HR = 1.143; 95% CI: 1.092, 1.197; p < 0.0001). On competing risk analysis, surviving patients with alcohol use disorder had a significantly increased hazard of cerebral ischemia (HR = 1.359; 95% CI: 1.209, 1.528; p < 0.0001) compared to surviving patients with no substance use disorders. Surviving patients with alcohol use disorder had significantly decreased hazards of vasospasm (HR = 0.851; 95% CI: 0.780, 0.929; p = 0.0003), newly-diagnosed hydrocephalus (HR = 0.748; 95% CI: 0.700, 0.799; p < 0.0001), and cerebral infarction (HR = 0.914; 95% CI: 0.870, 0.961; p = 0.0003).

### Differential Morbidity and Mortality by Alcohol Use Disorder

**Fig. 4:**
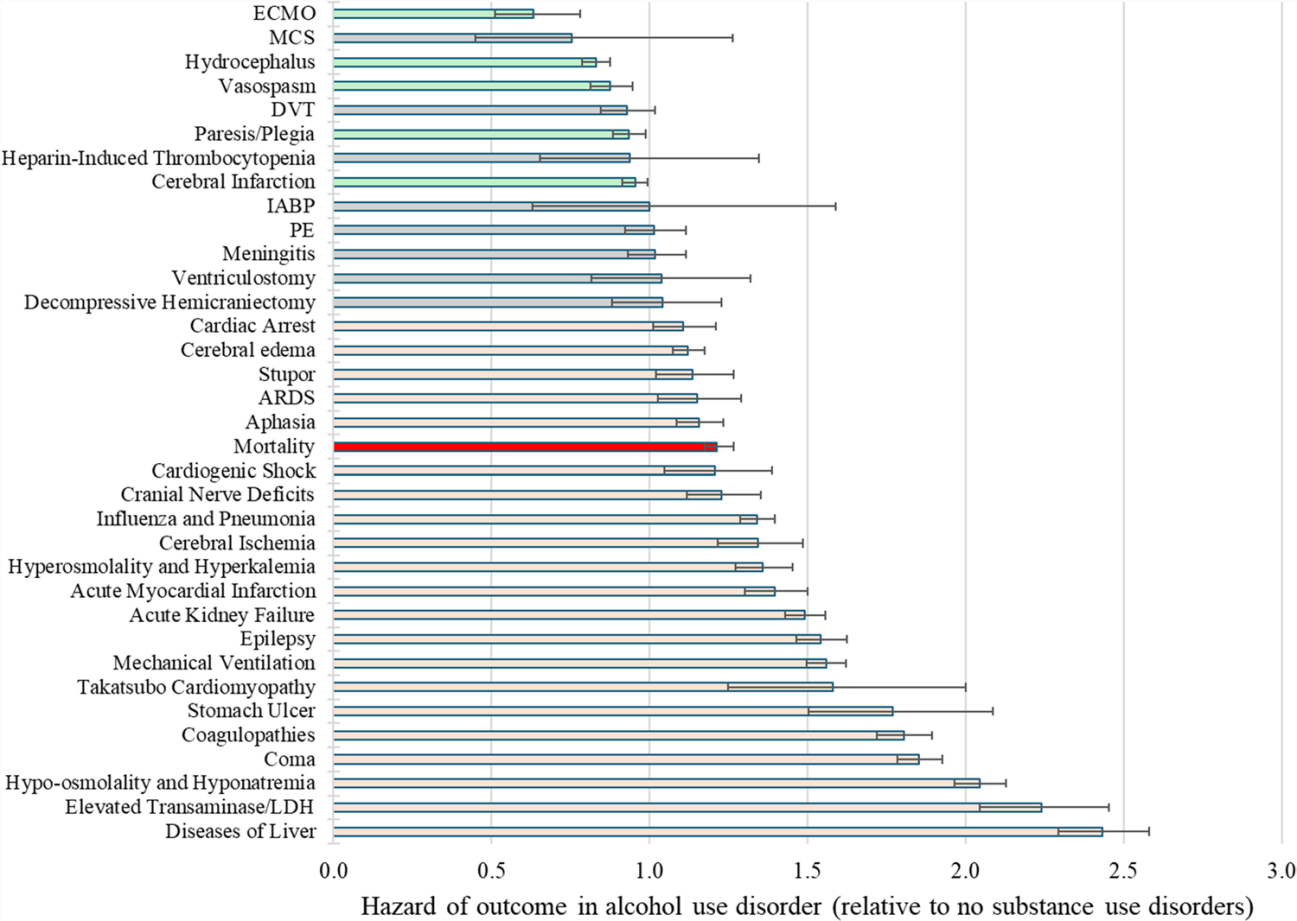
Adverse outcomes by alcohol use disorder status. Patients with alcohol use disorder were compared to patients with no substance use disorders using fully adjusted Cox proportional hazard models. Green indicates significantly decreased risk, grey indicates no significant effect, and light red indicates significantly elevated risk. Mortality is highlighted in bold red. Abbreviations are listed with Supplemental Table 3. Error bars indicate 95% confidence interval

Patients with alcohol use disorder had a greater burden of morbidity than patients with no substance use disorders (Fig. 4; full data shown in Supplemental Table 3). The greatest absolute differences in post-hemorrhage events were in hypo-osmolality and hyponatremia (25.7% vs. 13.6%; p < 0.0001), coma (28.1% vs. 16.0%; p < 0.0001), liver diseases (14.8% vs. 6.6%; p < 0.0001), and coagulopathies (18.2% vs. 10.7%; p < 0.0001). Other noteworthy differences in outcomes included cerebral edema (16.3% vs. 14.6%; p < 0.0001), epilepsy (14.0% vs. 9.5%; p < 0.0001), pneumonia (19.4% vs. 15.1%; p < 0.0001), and acute myocardial infarction (6.8% vs. 5.1%; p < 0.0001).

## Discussion

In this longitudinal, retrospective cohort study of over 216,000 patients with non-traumatic SAH, alcohol use at the time of SAH protected against mortality with a significant dose–response relationship. In contrast, alcohol use disorder was found to be associated with increased post-hemorrhage cerebral ischemia and mortality. Competing risk analysis showed that any apparent protective relationships likely reflected a survivorship bias and a shift in the severity of outcomes towards mortality. Even with this shift, the burden of morbidity among SAH survivors was even more devastating in patients with a history of pre-hemorrhage alcohol use disorder. To our knowledge, this is the first study of its kind to index patients based on SAH and study the role of alcohol use and alcohol use disorder in modulating post-hemorrhage outcomes.

### Clinical Outcomes of Alcohol Use at the Time of SAH

Many prior studies have attempted to discern the relationship between alcohol use and stroke mortality. While some demonstrated a J-shape curve (Protective at low dose, harmful at high dose), others showed a linear dose–response increase in mortality risk [2, 4, 19, 20, 25, 33, 41, 50, 65, 69]. Another cohort study showed increased risks of mortality and severe disability in heavy drinking [25]. Interestingly, our data showed a dose–response protective relationship. Alcohol use has been associated with increased high-density lipoprotein levels and acutely lowered blood pressure, particularly at mild-moderate levels [15]. Acute alcohol use may induce a hypercoagulable state mediated by increased factor VII, fibrinogen, and viscosity in both mild-moderate and heavy consumption [36, 54, 57]. Numerous studies have linked acute alcohol intake to elevation of plasminogen activator inhibitor-1 (PAI-1), the most potent inhibitor of fibrinolysis [11, 24, 42, 51, 66, 67]. The PAI-1 elevation appears to have a dose-dependent mechanism, with effects lasting longer in heavier alcohol use [24, 67]. This pro-coagulable state could potentially reduce sequelae of SAH in patients with recent alcohol use. Finally, mild-moderate alcohol use may involve enhanced nitric oxide production, which reduces cerebral vasospasm, and inhibition of platelet aggregation, which could mitigate secondary complications [40]. To this effect, multiple studies of intracranial aneurysms have demonstrated that current alcohol use is associated with rupture, while past alcohol use has no significant effects [5, 7]. Mild-moderate alcohol use has even been suggested to be neuroprotective [63].

### Clinical Outcomes of Alcohol Use Disorder in SAH

While alcohol use disorder is a known risk factor for SAH, few studies have investigated outcomes following hemorrhage [21, 27, 29, 30, 38, 53, 71]. Two previous cohort studies demonstrated that alcohol use disorder may increase the risk of vasospasm [55, 72]. In contrast, a systematic review showed no correlation between alcohol abuse and delayed cerebral ischemia, and neither did the two cohort studies [10, 25, 34]. Among SAH patients with alcohol use disorder, hemorrhagic factors that have been associated with worsening outcomes include the development of a new neurological defect, midline shift, and new-onset hydrocephalus [58]. The combination of both a neurological defect and hydrocephalus may be predictive of poor outcome, irrespective of age and midline shift [58]. These findings, in conjunction with those demonstrated in this study, highlight the constellation of signs that may be predictive in further identifying the most vulnerable patients with SAH.

### Limitations

This study is primarily limited by its retrospective, database methodology. All diagnoses, procedures, and laboratory tests were ascertained through the medical record, as opposed to patient- or physician-reported data. A limitation inherent to the TriNetX Advanced Analytics Platform is that updates and new patients are added daily, and healthcare organization servers are periodically unreachable, so the number of patients present at the moment of each individual analysis may fluctuate. Therefore, patient counts may have slight discrepancies across tables and figures, based on the moment of each analysis. The database is unable to provide granular information regarding specialty of consulting physician, which guidelines were followed, or what the severity of the condition was at the time of diagnosis [64]. In contrast, most existing studies have utilized questionnaires and patient interviews as part of large, prospective, longitudinal cohorts, although inherent limitations in these methods include sampling, recall, underreporting, severity, and survivorship biases [2, 4, 12, 19–23, 29, 36, 41, 50, 55–57, 59, 65, 68, 69]. Given the size of the network (approximately 141 million patients across 107 healthcare organizations in multiple countries), heterogeneity of clinical practice was inevitable between and within healthcare organizations, including missing data, differences in diagnostic criteria, insurance coverage, and inpatient vs. outpatient differences in coding and recording. While all non-traumatic subarachnoid hemorrhages were grouped together, prior studies using different methodologies (i.e., neither TriNetX specifically nor database studies generally) have reported that up to 15% of these are non-aneurysmal but were indistinguishable from the aneurysmal hemorrhages using ICD-10 codes, and it is possible that the true prevalence of non-aneurysmal SAH may have been even higher [1, 9, 28]. Head trauma may have been present but unreported due to memory impairment associated with either trauma or intoxication at the time of SAH. ICD-10 codes for alcohol use disorder (F10), vasospasm (G67.84), hydrocephalus (G91), cerebral infarction (I63), and cerebral ischemia (I67.82) may have wide variability in criteria and utilization leading to significant misclassification errors in both exposures and outcomes [39, 43]. Functional measures are not available for study in the TriNetX Research Network, so the NIS-SSS criteria were used as individual covariates [26, 32, 37, 55, 67]. The ICD-10 codes for “sequelae of non-traumatic subarachnoid hemorrhage” (I69.0**) contain extensive descriptions of potential cognitive and motor outcomes, but these were not recorded with any meaningful frequency. There was likely a selection bias in the patients who received blood alcohol testing, especially given that approximately 80% of patients had some level of alcohol in their blood on the day of hemorrhage. The acute effects of alcohol use may have had an unknown effect on clinicians’ assessments of SAH severity as assessed by NIS-SSS criteria, and this may not have been adequately accounted for by the inclusion of blood alcohol level at the time of SAH due to the infrequency of blood alcohol testing.

It is also possible that the apparent protective effect of alcohol on mortality could be subject to a survivorship bias in which patients with greater alcohol levels died before alcohol testing could be performed. Specifically, it has been suggested that patients who routinely drink more are likely to die prematurely, creating a “healthy survivor” bias [45]. Compounding this, younger individuals who consume alcohol are more likely to have higher incomes, educational achievement, and physical activity levels, coupled with fewer illnesses [17, 46, 48]. As 70% of binge drinking episodes occur in individuals who consume two or fewer drinks per day [47], the patients classified here as having the highest exposure to alcohol may routinely only imbibe low levels. The classification of patients as having no alcohol in their blood at the time of SAH subjects them to the “former drinker” bias, in which truly abstinent patients were combined with patients whose extensive past experience with alcohol persuaded them to stop drinking as well as patients who routinely consume alcohol but did not on the particular day of their hemorrhage [14]. In a systematic review with meta-analysis of 87 studies and 4,000,000 patients, accounting for this former drinker bias nullified the widely accepted J-shaped protective effect of moderate alcohol consumption and mortality [60].

## Conclusions

This longitudinal, retrospective cohort study found that alcohol use at the time of non-traumatic SAH was protective against mortality in a dose-dependent manner. In contrast, alcohol use disorder found to increase the hazards of mortality and cerebral ischemia following SAH. These findings are important for physicians to be aware of when identifying SAH patients at greatest risk of poor clinical outcomes. The established risk of SAH should be concurrently considered with the additional risks of adverse outcomes after SAH as part of counseling patients who have consumed alcohol and patients with alcohol use disorder. While this study is primarily limited by its retrospective, database methodology, which did not allow for patient interaction and radiographic analysis, prospective studies may be more equipped to overcome these restrictions.

## Supplementary Information

Below is the link to the electronic supplementary material.

Supplementary Material 1 (DOCX 28.5 KB)

Supplementary Material 2 (DOCX 29.3 KB)

Supplementary Material 3 (DOCX 36.8 KB)

## Data Availability

The data that support the findings of this study are available from TriNetX, LLC but third-party restrictions apply to the availability of these data. The data were used under license for this study with restrictions that do not allow for the data to be redistributed or made publicly available. However, for accredited researchers, the TriNetX data is available for licensing at TriNetX, LLC. Data access may require a data sharing agreement and may incur data access fees.

## References

[1] Bakker NA, Groen RJ, Foumani M, Uyttenboogaart M, Eshghi OS, Metzemaekers JD, Lammers N, Luijckx GJ, Van Dijk JM (2014) Repeat digital subtraction angiography after a negative baseline assessment in nonperimesencephalic subarachnoid hemorrhage: a pooled data meta-analysis. J Neurosurg 120:99–103. 10.3171/2013.9.Jns131337 24160474 10.3171/2013.9.JNS131337

[2] Bazzano LA, Gu D, Reynolds K, Wu X, Chen C-S, Duan X, Chen J, Wildman RP, Klag MJ, He J (2007) Alcohol consumption and risk for stroke among Chinese men. Ann Neurol 62:569–578. 10.1002/ana.21194 17708552 10.1002/ana.21194

[3] Bucci T, Choi SE, Tsang CT, Yiu KH, Buckley BJ, Pignatelli P, Scheitz JF, Lip GY, Abdul-Rahim AH (2024) Incident dementia in ischaemic stroke patients with early cardiac complications: a propensity-score matched cohort study. Eur Stroke J. 10.1177/23969873241293573 39487764 10.1177/23969873241293573 PMC11558657

[4] Camargo CA Jr, Hennekens CH, Gaziano JM, Glynn RJ, Manson JE, Stampfer MJ (1997) Prospective study of moderate alcohol consumption and mortality in US male physicians. Arch Intern Med 157:79–85. 10.1001/archinte.1997.00440220083011 8996044

[5] Can A, Castro VM, Ozdemir YH, Dagen S, Dligach D, Finan S, Yu S, Gainer V, Shadick NA, Savova G, Murphy S, Cai T, Weiss ST, Du R (2018) Alcohol consumption and aneurysmal subarachnoid hemorrhage. Transl Stroke Res 9:13–19. 10.1007/s12975-017-0557-z 28752411 10.1007/s12975-017-0557-z

[6] Caplan LR (2008) Dissections of brain-supplying arteries. Nat Clin Pract Neurol 4:34–42. 10.1038/ncpneuro0683 18199995 10.1038/ncpneuro0683

[7] Chen C-J, Brown WM, Moomaw CJ, Langefeld CD, Osborne J, Worrall BB, Woo D, Koch S, For the EI (2017) Alcohol use and risk of intracerebral hemorrhage. Neurology 88:2043–2051. 10.1212/WNL.0000000000003952 28446657 10.1212/WNL.0000000000003952 PMC5440244

[8] Claassen J, Park S (2022) Spontaneous subarachnoid haemorrhage. Lancet 400:846–862. 10.1016/s0140-6736(22)00938-2 35985353 10.1016/S0140-6736(22)00938-2 PMC9987649

[9] Delgado Almandoz JE, Jagadeesan BD, Refai D, Moran CJ, Cross DT 3rd, Chicoine MR, Rich KM, Diringer MN, Dacey RG Jr, Derdeyn CP, Zipfel GJ (2012) Diagnostic yield of repeat catheter angiography in patients with catheter and computed tomography angiography negative subarachnoid hemorrhage. Neurosurgery 70:1135–1142. 10.1227/NEU.0b013e318242575e 22105208 10.1227/NEU.0b013e318242575e

[10] de Rooij NK, Rinkel GJ, Dankbaar JW, Frijns CJ (2013) Delayed cerebral ischemia after subarachnoid hemorrhage: a systematic review of clinical, laboratory, and radiological predictors. Stroke 44:43–54. 10.1161/strokeaha.112.674291 23250997 10.1161/STROKEAHA.112.674291

[11] Dimmitt S, Rakic V, Puddey I, Baker R, Oostryck R, Adams M, Chesterman C, Burke V, Beilin L (1998) The effects of alcohol on coagulation and fibrinolytic factors: a controlled trial. Blood Coagul Fibrinolysis 9:39–46 9607117 10.1097/00001721-199801000-00005

[12] Donahue RP, Abbott RD, Reed DM, Yano K (1986) Alcohol and hemorrhagic stroke. The Honolulu Heart Program. JAMA 255:2311–2314 3959320

[13] Etminan N, Chang HS, Hackenberg K, de Rooij NK, Vergouwen MDI, Rinkel GJE, Algra A (2019) Worldwide incidence of aneurysmal subarachnoid hemorrhage according to region, time period, blood pressure, and smoking prevalence in the population: a systematic review and meta-analysis. JAMA Neurol 76:588–597. 10.1001/jamaneurol.2019.0006 30659573 10.1001/jamaneurol.2019.0006 PMC6515606

[14] Fillmore KM, Stockwell T, Chikritzhs T, Bostrom A, Kerr W (2007) Moderate alcohol use and reduced mortality risk: systematic error in prospective studies and new hypotheses. Ann Epidemiol 17:S16–S23. 10.1016/j.annepidem.2007.01.005 17478320 10.1016/j.annepidem.2007.01.005

[15] Gaziano JM, Buring JE, Breslow JL, Goldhaber SZ, Rosner B, VanDenburgh M, Willett W, Hennekens CH (1993) Moderate alcohol intake, increased levels of high-density lipoprotein and its subfractions, and decreased risk of myocardial infarction. N Engl J Med 329:1829–1834. 10.1056/NEJM199312163292501 8247033 10.1056/NEJM199312163292501

[16] Gill JS, Shipley MJ, Tsementzis SA, Hornby RS, Gill SK, Hitchcock ER, Beevers DG (1991) Alcohol consumption–a risk factor for hemorrhagic and non-hemorrhagic stroke. Am J Med 90:489–497 2012089

[17] Green CA, Polen MR (2001) The health and health behaviors of people who do not drink alcohol. Am J Prev Med 21:298–305. 10.1016/s0749-3797(01)00365-8 11701301 10.1016/s0749-3797(01)00365-8

[18] Hanel RA, Xavier AR, Mohammad Y, Kirmani JF, Yahia AM, Qureshi AI (2002) Outcome following intracerebral hemorrhage and subarachnoid hemorrhage. Neurol Res 24:58–62. 10.1179/016164102101200041 10.1179/016164102101200041 12074438

[19] Hansagi H, Romelsjö A, Gerhardsson de Verdier M, Andréasson S, Leifman A (1995) Alcohol consumption and stroke mortality. 20-year follow-up of 15,077 men and women. Stroke 26:1768–1773. 10.1161/01.str.26.10.1768 7570723 10.1161/01.str.26.10.1768

[20] Hart CL, Davey Smith G, Hole DJ, Hawthorne VM (1999) Alcohol consumption and mortality from all causes, coronary heart disease, and stroke: results from a prospective cohort study of Scottish men with 21 years of follow up. BMJ 318:1725–1729. 10.1136/bmj.318.7200.1725 10381706 10.1136/bmj.318.7200.1725 PMC31100

[21] Ikehara S, Iso H, Yamagishi K, Kokubo Y, Saito I, Yatsuya H, Inoue M, Tsugane S (2013) Alcohol consumption and risk of stroke and coronary heart disease among Japanese women: the Japan Public Health Center-based prospective study. Prev Med 57:505–510. 10.1016/j.ypmed.2013.07.003 23859928 10.1016/j.ypmed.2013.07.003

[22] Iso H, Baba S, Mannami T, Sasaki S, Okada K, Konishi M, Tsugane S (2004) Alcohol consumption and risk of stroke among middle-aged men: the JPHC study cohort I. Stroke 35:1124–1129. 10.1161/01.Str.0000124459.33597.00 15017008 10.1161/01.STR.0000124459.33597.00

[23] Iso H, Kitamura A, Shimamoto T, Sankai T, Naito Y, Sato S, Kiyama M, Iida M, Komachi Y (1995) Alcohol intake and the risk of cardiovascular disease in middle-aged Japanese men. Stroke 26:767–773. 10.1161/01.str.26.5.767 7740564 10.1161/01.str.26.5.767

[24] Johansen KM, Skorpe S, Olsen JO, Østerud B (1999) The effect of red wine on the fibrinolytic system and the cellular activation reactions before and after exercise. Thromb Res 96:355–363 10605950 10.1016/s0049-3848(99)00120-6

[25] Juvela S (1992) Alcohol consumption as a risk factor for poor outcome after aneurysmal subarachnoid haemorrhage. BMJ 304:1663–1667. 10.1136/bmj.304.6843.1663 1633519 10.1136/bmj.304.6843.1663 PMC1882384

[26] Khan F, Peyvandi F, Clare K, Nolan B, Patel S, Feldstein E, Ogulnick JV, Said A, Zeller S, Bornovski Y, Wong S, Medicherla CB, Rosenberg J, Miller D, Coritsidis G, Prabhakaran K, Mayer SA, Gandhi CD, Al-Mufti F (2023) Aneurysmal Subarachnoid Hemorrhage and Cardiac Related Fatality: Who Dies and Why? Cardiology. 10.1097/CRD.0000000000000568. (**in Review**) 37432015 10.1097/CRD.0000000000000568

[27] Klatsky AL, Armstrong MA, Friedman GD, Sidney S (2002) Alcohol drinking and risk of hemorrhagic stroke. Neuroepidemiology 21:115–122. 10.1159/000054808 12006774 10.1159/000054808

[28] Konczalla J, Platz J, Schuss P, Vatter H, Seifert V, Güresir E (2014) Non-aneurysmal non-traumatic subarachnoid hemorrhage: patient characteristics, clinical outcome and prognostic factors based on a single-center experience in 125 patients. BMC Neurol 14:140. 10.1186/1471-2377-14-140 24986457 10.1186/1471-2377-14-140 PMC4088361

[29] Korja M, Silventoinen K, Laatikainen T, Jousilahti P, Salomaa V, Hernesniemi J, Kaprio J (2013) Risk factors and their combined effects on the incidence rate of subarachnoid hemorrhage–a population-based cohort study. PLoS One 8:e73760. 10.1371/journal.pone.0073760 24040058 10.1371/journal.pone.0073760 PMC3767622

[30] Kubota M, Yamaura A, Ono J (2001) Prevalence of risk factors for aneurysmal subarachnoid haemorrhage: results of a Japanese multicentre case control study for stroke. Br J Neurosurg 15:474–478. 10.1080/02688690120097697 11813998 10.1080/02688690120097697

[31] Kumar S, Goddeau RP Jr, Selim MH, Thomas A, Schlaug G, Alhazzani A, Searls DE, Caplan LR (2010) Atraumatic convexal subarachnoid hemorrhage: clinical presentation, imaging patterns, and etiologies. Neurology 74:893–899. 10.1212/WNL.0b013e3181d55efa 20231664 10.1212/WNL.0b013e3181d55efa PMC2836868

[32] Lantigua H, Ortega-Gutierrez S, Schmidt JM, Lee K, Badjatia N, Agarwal S, Claassen J, Connolly ES, Mayer SA (2015) Subarachnoid hemorrhage: who dies, and why? Crit Care 19:309. 10.1186/s13054-015-1036-0 26330064 10.1186/s13054-015-1036-0 PMC4556224

[33] Larsson SC, Wallin A, Wolk A, Markus HS (2016) Differing association of alcohol consumption with different stroke types: a systematic review and meta-analysis. BMC Med 14:178. 10.1186/s12916-016-0721-4 27881167 10.1186/s12916-016-0721-4 PMC5121939

[34] Lasner TM, Weil RJ, Riina HA, King JT Jr., Zager EL, Raps EC, Flamm ES (1997) Cigarette smoking-induced increase in the risk of symptomatic vasospasm after aneurysmal subarachnoid hemorrhage. J Neurosurg 87:381–384. 10.3171/jns.1997.87.3.0381 10.3171/jns.1997.87.3.0381 9285602

[35] Laug WE (1983) Ethyl alcohol enhances plasminogen activator secretion by endothelial cells. JAMA 250:772–776 6683764

[36] Lee KW, Lip GYH (2003) Effects of lifestyle on hemostasis, fibrinolysis, and platelet reactivity: a systematic review. Arch Intern Med 163:2368–2392. 10.1001/archinte.163.19.2368 14581258 10.1001/archinte.163.19.2368

[37] Lee HS, Sohn MK, Lee J, Kim DY, Shin Y-I, Oh G-J, Lee Y-S, Joo MC, Lee SY, Song M-K, Han J, Ahn J, Lee Y-H, Kim DH, Kim Y-T, Kim Y-H, Chang WH (2025) Five-year functional outcomes among patients surviving aneurysmal subarachnoid hemorrhage. JAMA Netw Open 8:e251678–e251678. 10.1001/jamanetworkopen.2025.1678 40131277 10.1001/jamanetworkopen.2025.1678 PMC11937949

[38] Longstreth WT Jr, Nelson LM, Koepsell TD, van Belle G (1992) Cigarette smoking, alcohol use, and subarachnoid hemorrhage. Stroke 23:1242–1249. 10.1161/01.str.23.9.1242 1519278 10.1161/01.str.23.9.1242

[39] Lundin A, Hallgren M, Forsman M, Forsell Y (2015) Comparison of DSM-5 classifications of alcohol use disorders with those of DSM-IV, DSM-III-R, and ICD-10 in a general population sample in Sweden. J Stud Alcohol Drugs 76:773–780. 10.15288/jsad.2015.76.773 26402358 10.15288/jsad.2015.76.773

[40] Martin S, Diebolt M, Andriantsitohaina R (2001) Moderate alcohol consumption and cardiovascular diseases. Pathol Biol 49:769–774. 10.1016/s0369-8114(01)00241-3 11762141 10.1016/s0369-8114(01)00241-3

[41] Maskarinec G, Meng L, Kolonel LN (1998) Alcohol intake, body weight, and mortality in a multiethnic prospective cohort. Epidemiology 9:654–661 9799177

[42] McConnell MV, Vavouranakis I, Wu LL, Vaughan DE, Ridker PM (1997) Effects of a single, daily alcoholic beverage on lipid and hemostatic markers of cardiovascular risk. Am J Cardiol 80:1226–1228 9359559 10.1016/s0002-9149(97)00647-4

[43] Milan JB, Jensen TSR, Nørager N, Pedersen SSH, Riedel CS, Toft NM, Ammar A, Foroughi M, Grotenhuis A, Perera A, Rekate H, Juhler M (2023) The ASPECT hydrocephalus system: a non-hierarchical descriptive system for clinical use. Acta Neurochir (Wien) 165:355–365. 10.1007/s00701-022-05412-6 36427098 10.1007/s00701-022-05412-6 PMC9922243

[44] Moncada S, Radomski MW (1985) The problems and the promise of prostaglandin influences in atherogenesis. Ann N Y Acad Sci 454:121–130. 10.1111/j.1749-6632.1985.tb11850.x 3935029 10.1111/j.1749-6632.1985.tb11850.x

[45] Naimi TS, Brewer RD, Mokdad A, Denny C, Serdula MK, Marks JS (2003) Binge drinking among US adults. JAMA 289:70–75. 10.1001/jama.289.1.70 12503979 10.1001/jama.289.1.70

[46] Naimi TS, Brown DW, Brewer RD, Giles WH, Mensah G, Serdula MK, Mokdad AH, Hungerford DW, Lando J, Naimi S, Stroup DF (2005) Cardiovascular risk factors and confounders among nondrinking and moderate-drinking U.S. adults. Am J Prev Med 28:369–373. 10.1016/j.amepre.2005.01.011 15831343 10.1016/j.amepre.2005.01.011

[47] Naimi TS, Stockwell T, Zhao J, Xuan Z, Dangardt F, Saitz R, Liang W, Chikritzhs T (2017) Selection biases in observational studies affect associations between ‘moderate’ alcohol consumption and mortality. Addiction 112:207–214. 10.1111/add.13451 27316346 10.1111/add.13451

[48] Ng Fat L, Shelton N (2012) Associations between self-reported illness and non-drinking in young adults. Addiction 107:1612–1620. 10.1111/j.1360-0443.2012.03878.x 22404244 10.1111/j.1360-0443.2012.03878.x

[49] Palchuk MB, London JW, Perez-Rey D, Drebert ZJ, Winer-Jones JP, Thompson CN, Esposito J, Claerhout B (2023) A global federated real-world data and analytics platform for research. JAMIA Open 6:ooad035. 10.1093/jamiaopen/ooad035 37193038 10.1093/jamiaopen/ooad035 PMC10182857

[50] Palmer AJ, Fletcher AE, Bulpitt CJ, Beevers DG, Coles EC, Ledingham JGG, Petrie JC, Webster J, Dollery CT (1995) Alcohol intake and cardiovascular mortality in hypertensive patients: report from the department of health hypertension care computing project. J Hypertens. 10.1097/00004872-199509000-00004 8586830 10.1097/00004872-199509000-00004

[51] Pellegrini N, Pareti F, Stabile F, Brusamolino A, Simonetti P (1996) Effects of moderate consumption of red wine on platelet aggregation and haemostatic variables in healthy volunteers. Eur J Clin Nutr 50:209–213 8730606

[52] Rautalin IM, Asikainen A, Korja M (2024) Modifiable risk factors for subarachnoid hemorrhage: narrative review with an emphasis on common controversies and epidemiologic pitfalls. Neurology 103:e210052. 10.1212/wnl.0000000000210052 39556778 10.1212/WNL.0000000000210052 PMC11627175

[53] Reynolds K, Lewis B, Nolen JD, Kinney GL, Sathya B, He J (2003) Alcohol consumption and risk of stroke: a meta-analysis. JAMA 289:579–588. 10.1001/jama.289.5.579 12578491 10.1001/jama.289.5.579

[54] Ridker PM, Hennekens CH, Manson JE, Ridker PM, Vaughan DE, Stampfer MJ, Vaughan DE, Hennekens CH, Stampfer MJ (1994) Prospective study of endogenous tissue plasminogen activator and risk of stroke. Lancet 343:940–943. 10.1016/S0140-6736(94)90064-7 7909008 10.1016/s0140-6736(94)90064-7

[55] Rumalla K, Lin M, Ding L, Gaddis M, Giannotta SL, Attenello FJ, Mack WJ (2021) Risk factors for cerebral vasospasm in aneurysmal subarachnoid hemorrhage: a population-based study of 8346 patients. World Neurosurg 145:e233–e241. 10.1016/j.wneu.2020.10.008 33049382 10.1016/j.wneu.2020.10.008

[56] Sankai T, Iso H, Shimamoto T, Kitamura A, Naito Y, Sato S, Okamura T, Imano H, Iida M, Komachi Y (2000) Prospective study on alcohol intake and risk of subarachnoid hemorrhage among Japanese men and women. Alcohol Clin Exp Res 24:386–389 10776682

[57] Smith FB, Lee AJ, Fowkes FG, Price JF, Rumley A, Lowe GD (1997) Hemostatic factors as predictors of ischemic heart disease and stroke in the Edinburgh Artery Study. Arterioscler Thromb Vasc Biol 17:3321–3325. 10.1161/01.atv.17.11.3321 9409328 10.1161/01.atv.17.11.3321

[58] Spille DC, Kuroczik D, Görlich D, Varghese J, Schwake M, Stummer W, Holling M (2024) Which risk factors significantly influence the outcome of traumatic brain injured patients with alcohol use disorder? Eur J Trauma Emerg Surg 50:1187–1197. 10.1007/s00068-023-02346-1 37578515 10.1007/s00068-023-02346-1 PMC11458655

[59] Stampfer MJ, Colditz GA, Willett WC, Speizer FE, Hennekens CH (1988) A prospective study of moderate alcohol consumption and the risk of coronary disease and stroke in women. N Engl J Med 319:267–273. 10.1056/nejm198808043190503 3393181 10.1056/NEJM198808043190503

[60] Stockwell T, Zhao J, Panwar S, Roemer A, Naimi T, Chikritzhs T (2016) Do “Moderate” Drinkers Have Reduced Mortality Risk? A Systematic Review and Meta-Analysis of Alcohol Consumption and All-Cause Mortality. J Stud Alcohol Drugs 77:185–198. 10.15288/jsad.2016.77.185 26997174 10.15288/jsad.2016.77.185 PMC4803651

[61] Stokes GS (1982) Hypertension and alcohol: is there a link? J Chronic Dis 35:759–762. 10.1016/0021-9681(82)90086-8 7119077 10.1016/0021-9681(82)90086-8

[62] Tatter SB, Crowell RM, Ogilvy CS (1995) Aneurysmal and microaneurysmal “angiogram-negative” subarachnoid hemorrhage. Neurosurgery 37:48–55. 10.1227/00006123-199507000-00007 8587690 10.1227/00006123-199507000-00007

[63] Thangameeran SI, Wang P-K, Liew H-K, Pang C-Y (2024) Influence of alcohol on intracerebral hemorrhage: from oxidative stress to glial cell activation. Life 14(3):311. 10.3390/life14030311 38541637 10.3390/life14030311 PMC10971394

[64] Thorman IB, Schrack JA, Schubert MC (2024) Epidemiology and comorbidities of vestibular disorders: preliminary findings of the AVOCADO study. Otol Neurotol 45:572–579. 10.1097/mao.0000000000004185 38728561 10.1097/MAO.0000000000004185

[65] Thun MJ, Peto R, Lopez AD, Monaco JH, Henley SJ, Heath CW, Doll R (1997) Alcohol consumption and mortality among middle-aged and elderly U.S. adults. N Engl J Med 337:1705–1714. 10.1056/NEJM199712113372401 9392695 10.1056/NEJM199712113372401

[66] Van de Wiel A, Van Golde P, Kraaijenhagen R, Von dem Borne P, Bouma B, Hart H (2001) Acute inhibitory effect of alcohol on fibrinolysis. Eur J Clin Invest 31:164–170 11168456 10.1046/j.1365-2362.2001.00773.x

[67] Washington CW, Derdeyn CP, Dacey RG, Dhar R, Zipfel GJ (2014) Analysis of subarachnoid hemorrhage using the Nationwide Inpatient Sample: the NIS-SAH severity score and outcome measure: clinical article. J Neurosurg 121:482–489. 10.3171/2014.4.JNS131100 24949676 10.3171/2014.4.JNS131100

[68] Yamada S, Koizumi A, Iso H, Wada Y, Watanabe Y, Date C, Yamamoto A, Kikuchi S, Inaba Y, Toyoshima H, Kondo T, Tamakoshi A (2003) Risk factors for fatal subarachnoid hemorrhage: the Japan collaborative cohort study. Stroke 34:2781–2787. 10.1161/01.Str.0000103857.13812.9a 14657543 10.1161/01.STR.0000103857.13812.9A

[69] Yang L, Zhou M, Sherliker P, Cai Y, Peto R, Wang L, Millwood I, Smith M, Hu Y, Yang G, Chen Z (2012) Alcohol drinking and overall and cause-specific mortality in China: nationally representative prospective study of 220 000 men with 15 years of follow-up. Int J Epidemiol 41:1101–1113. 10.1093/ije/dys075 22596929 10.1093/ije/dys075

[70] Yao X, Zhang K, Bian J, Chen G (2016) Alcohol consumption and risk of subarachnoid hemorrhage: a meta-analysis of 14 observational studies. Biomed Rep 5:428–436. 10.3892/br.2016.743 27699009 10.3892/br.2016.743 PMC5038345

[71] Zhang C, Qin Y-Y, Chen Q, Jiang H, Chen X-Z, Xu C-L, Mao P-J, He J, Zhou Y-H (2014) Alcohol intake and risk of stroke: a dose–response meta-analysis of prospective studies. Int J Cardiol 174:669–677. 10.1016/j.ijcard.2014.04.225 24820756 10.1016/j.ijcard.2014.04.225

[72] Zhao L, Cheng C, Peng L, Zuo W, Xiong D, Zhang L, Mao Z, Zhang J, Wu X, Jiang X, Wang P, Li W (2022) Alcohol abuse associated with increased risk of angiographic vasospasm and delayed cerebral ischemia in patients with aneurysmal subarachnoid hemorrhage requiring mechanical ventilation. Front Cardiovasc Med 9:825890. 10.3389/fcvm.2022.825890 35620515 10.3389/fcvm.2022.825890 PMC9127604

