# Supplementary material for: Impact of acute alcohol intoxication and alcohol dependence on outcomes after subarachnoid hemorrhage
Source: Acta Neurochir (Wien). 2025 Aug 27;167(1):231. doi: 10.1007/s00701-025-06639-9 (PMC12390883; doi:10.1007/s00701-025-06639-9)
Supplement: Supplementary file 1 — Supplementary Material 1 (DOCX 28.5 KB) [file 701_2025_6639_MOESM1_ESM.docx]

**Supplemental Table 1**. Code Mapping for Laboratory Test Matching

| **Code Type** | **Code** | **Description** |
| --- | --- | --- |
| TriNetX Curated | 9032 | International Normalized Ratio (INR) in plasma or blood |
| TriNetX Curated | 9033 | Prothrombin Time (PT) in plasma or blood |
| TriNetX Curated | 9031 | Activated Partial Thromboplastin Time (aPTT) in plasma or blood |
| TriNetX Curated | LG2847-4 | Amphetamines [presence] in urine |
| LOINC | 3397-7 | Cocaine [presence] in urine |
| TriNetX Curated | LG7013-8 | Opiates [presence] in urine |
| LOINC | 19659-2 | Phencyclidine [presence] in urine by screen method |
| LOINC | 18282-4 | Cannabinoids [presence] in urine by screen method |
| TriNetX Curated | LG2-3604 | Ethanol [mass/volume] in serum, plasma, or blood |
