# Supplementary material for: Impact of acute alcohol intoxication and alcohol dependence on outcomes after subarachnoid hemorrhage
Source: Acta Neurochir (Wien). 2025 Aug 27;167(1):231. doi: 10.1007/s00701-025-06639-9 (PMC12390883; doi:10.1007/s00701-025-06639-9)
Supplement: Supplementary file 2 — Supplementary Material 2 (DOCX 29.3 KB) [file 701_2025_6639_MOESM2_ESM.docx]

**Supplemental Table 2**. Diagnosis and Procedure Codes for NIS-SSS

| **Code** | **Diagnosis Description** | **Code** | **Procedure Description** |
| --- | --- | --- | --- |
| G91 | Hydrocephalus | 02.2 | Ventriculostomy |
| H49 | Paralytic strabismus | 0016070 | Bypass cerebral ventricle to nasopharynx with autologous tissue substitute, open approach |
| H57.0 | Anomalies of pupillary function | 00163J2 | Bypass cerebral ventricle to atrium with synthetic substitute, percutaneous approach |
| I69 | Sequelae of cerebrovascular disease | 00160J4 | Bypass cerebral ventricle to pleural cavity with synthetic substitute, open approach |
| I69.92 | Speech and language deficits following unspecified cerebrovascular disease | 00163J6 | Bypass cerebral ventricle to peritoneal cavity with synthetic substitute, percutaneous approach |
| I69.93 | Monoplegia of upper limb following unspecified cerebrovascular disease | 0016077 | Bypass cerebral ventricle to urinary tract with autologous tissue substitute, open approach |
| I69.94 | Monoplegia of lower limb following unspecified cerebrovascular disease | 0016078 | Bypass cerebral ventricle to bone marrow with autologous tissue substitute, open approach |
| I69.95 | Hemiplegia and hemiparesis following unspecified cerebrovascular disease | 0BH17EZ | Insertion of endotracheal airway into trachea, via natural or artificial opening |
| I69.96 | Other paralytic syndrome following unspecified cerebrovascular disease | 5A19 | Physiological systems/performance/respiratory |
| I69.998 | Other sequelae following unspecified cerebrovascular disease | 5A1945Z | Respiratory ventilation, 24-96 consecutive hours |
| R40.1 | Stupor | 5A1955Z | Respiratory ventilation, greater than 96 consecutive hours |
| R40.2 | Coma |  |  |
