# Supplementary material for: Impact of acute alcohol intoxication and alcohol dependence on outcomes after subarachnoid hemorrhage
Source: Acta Neurochir (Wien). 2025 Aug 27;167(1):231. doi: 10.1007/s00701-025-06639-9 (PMC12390883; doi:10.1007/s00701-025-06639-9)
Supplement: Supplementary file 3 — Supplementary Material 3 (DOCX 36.8 KB) [file 701_2025_6639_MOESM3_ESM.docx]

**Supplemental Table 3.** Prevalence and Hazard of Complications With and Without Alcohol Use Disorder after SAH

*Excludes prevalent cases. ECMO: extracorporeal membrane oxygenation; IABP: intra-aortic balloon pump; MCS: mechanical cardiac support; LVAD: left ventricular assist device; ARDS: acute respiratory distress syndrome; LDH: lactate dehydrogenase; PE: pulmonary embolism; DVT: deep vein thrombosis.

| Condition | Alcohol Use Disorder | | | No Substance Use Disorders | | | Hazard with Alcohol Use Disorder | | | |
| --- | --- | --- | --- | --- | --- | --- | --- | --- | --- | --- |
|  | Patients | Outcome | % | Patients | Outcome | % | HR | CI Low | CI High | P-value |
| Mortality | 26,443 | 5,693 | 21.5% | 26,443 | 4,903 | 18.5% | 1.212 | 1.173 | 1.267 | **<0.0001** |
| Vasospasm | 26,443 | 1,233 | 4.7% | 26,443 | 1,399 | 5.3% | 0.876 | 0.812 | 0.946 | **0.0006** |
| Hydrocephalus* | 25,720 | 2,556 | 9.9% | 25,906 | 3,086 | 11.9% | 0.831 | 0.788 | 0.875 | **<0.0001** |
| Cerebral Infarction | 26,443 | 4,623 | 17.5% | 26,443 | 4,859 | 18.4% | 0.955 | 0.914 | 0.995 | **0.0229** |
| Cerebral Ischemia | 26,443 | 899 | 3.4% | 26,443 | 696 | 2.6% | 1.344 | 1.217 | 1.484 | **<0.0001** |
| Meningitis* | 26,043 | 965 | 3.7% | 26,166 | 955 | 3.6% | 1.018 | 0.931 | 1.114 | 0.6906 |
| Ventriculostomy* | 26,412 | 136 | 0.5% | 26,431 | 131 | 0.5% | 1.037 | 0.816 | 1.319 | 0.7641 |
| Decompressive Hemicraniectomy* | 26,351 | 283 | 1.1% | 26,384 | 271 | 1.0% | 1.040 | 0.880 | 1.228 | 0.7140 |
| Coma* | 24,299 | 6,838 | 28.1% | 25,501 | 4,079 | 16.0% | 1.853 | 1.783 | 1.927 | **<0.0001** |
| Stupor* | 25,684 | 701 | 2.7% | 26,169 | 632 | 2.4% | 1.136 | 1.020 | 1.265 | **0.0201** |
| Cerebral Edema* | 25,214 | 4,101 | 16.3% | 25,503 | 3,724 | 14.6% | 1.122 | 1.074 | 1.173 | **<0.0001** |
| Epilepsy* | 24,352 | 3,406 | 14.0% | 25,417 | 2,418 | 9.5% | 1.542 | 1.464 | 1.625 | **<0.0001** |
| Cranial Nerve Deficits* | 26,088 | 922 | 3.5% | 26,226 | 766 | 2.9% | 1.229 | 1.117 | 1.353 | **<0.0001** |
| Aphasia* | 25,617 | 1,989 | 7.8% | 25,892 | 1,765 | 6.8% | 1.158 | 1.086 | 1.234 | **<0.0001** |
| Paresis/Plegia* | 25,346 | 2,379 | 9.4% | 25,712 | 2,598 | 10.1% | 0.935 | 0.884 | 0.988 | **0.0169** |
| Takatsubo Cardiomyopathy* | 26,371 | 176 | 0.7% | 26,424 | 114 | 0.4% | 1.581 | 1.249 | 2.002 | **0.0001** |
| Cardiogenic Shock* | 26,196 | 424 | 1.6% | 26,271 | 363 | 1.4% | 1.206 | 1.048 | 1.388 | **0.0087** |
| Acute Myocardial Infarction | 26,443 | 1,796 | 6.8% | 26,443 | 1,351 | 5.1% | 1.397 | 1.301 | 1.499 | **<0.0001** |
| Cardiac Arrest | 26,443 | 1,021 | 3.9% | 26,443 | 944 | 3.6% | 1.107 | 1.013 | 1.209 | **0.0235** |
| ECMO* | 26,196 | 140 | 0.5% | 26,218 | 227 | 0.9% | 0.632 | 0.512 | 0.781 | **<0.0001** |
| IABP* | 26,393 | 35 | 0.1% | 26,408 | 37 | 0.1% | 1.000 | 0.629 | 1.589 | 0.9987 |
| MCS* | 26,408 | 25 | 0.1% | 26,396 | 34 | 0.1% | 0.754 | 0.449 | 1.264 | 0.2823 |
| LVAD* | 26,385 | ≤10 | -- | 26,386 | ≤10 | -- | 1.021 | 0.404 | 2.580 | 0.9657 |
| ARDS | 26,443 | 637 | 2.4% | 26,443 | 561 | 2.1% | 1.150 | 1.027 | 1.289 | **0.0151** |
| Influenza and Pneumonia | 26,443 | 5,126 | 19.4% | 26,443 | 4,004 | 15.1% | 1.341 | 1.286 | 1.397 | **<0.0001** |
| Mechanical Ventilation* | 24,139 | 5,792 | 24.0% | 25,298 | 4,067 | 16.1% | 1.558 | 1.497 | 1.622 | **<0.0001** |
| Stomach Ulcer* | 25,980 | 376 | 1.4% | 26,278 | 233 | 0.9% | 1.771 | 1.503 | 2.087 | **<0.0001** |
| Diseases of Liver* | 22,175 | 3,274 | 14.8% | 25,304 | 1,675 | 6.6% | 2.432 | 2.293 | 2.580 | **<0.0001** |
| Elevated Transaminase/LDH* | 25,096 | 1,397 | 5.6% | 26,070 | 700 | 2.7% | 2.241 | 2.046 | 2.455 | **<0.0001** |
| Hypo-osmolality and Hyponatremia | 26,443 | 6,824 | 25.8% | 26,443 | 3,606 | 13.6% | 2.045 | 1.964 | 2.129 | **<0.0001** |
| Hyperosmolality and Hyperkalemia | 26,443 | 1,957 | 7.4% | 26,443 | 1,512 | 5.7% | 1.359 | 1.271 | 1.454 | **<0.0001** |
| Acute Kidney Failure | 26,443 | 5,270 | 19.9% | 26,443 | 3,738 | 14.1% | 1.491 | 1.429 | 1.555 | **<0.0001** |
| Coagulopathies* | 22,967 | 4,181 | 18.2% | 24,990 | 2,669 | 10.7% | 1.805 | 1.719 | 1.895 | **<0.0001** |
| PE* | 25,790 | 854 | 3.3% | 26,022 | 870 | 3.3% | 1.015 | 0.923 | 1.116 | 0.7585 |
| DVT* | 25,855 | 884 | 3.4% | 26,017 | 973 | 3.7% | 0.928 | 0.847 | 1.017 | 0.1094 |
| Heparin-Induced Thrombocytopenia* | 26,417 | 57 | 0.2% | 26,425 | 61 | 0.2% | 0.939 | 0.654 | 1.347 | 0.7313 |
